# The Design of a Biomimetic Hierarchical Thin-Walled Structure Inspired by a Lotus Leaf and Its Mechanical Performance Analysis

**DOI:** 10.3390/ma16114116

**Published:** 2023-05-31

**Authors:** Lili Liu, Longhai Li, Ce Guo, Yizheng Ge, Yue Chen, Lei Zhang

**Affiliations:** 1 School of Mechanical and Electrical Engineering, Xuzhou University of Technology, Xuzhou 221018, China; lily_cumt2010@163.com (L.L.); gyz2312001215@163.com (Y.G.); chenyue@xzit.edu.cn (Y.C.); triple-stone@foxmail.com (L.Z.); 2 Institute of Bio-Inspired Structure and Surface Engineering, Nanjing University of Aeronautics and Astronautics, 29 Yudao Street, Nanjing 210016, China; guozc@nuaa.edu.cn

**Keywords:** biomimetic hierarchical thin-walled structures, finite element model, mechanical properties, energy absorption capacity

## Abstract

Inspired by the macro- and microstructures of the lotus leaf, a series of biomimetic hierarchical thin-walled structures (BHTSs) was proposed and fabricated, exhibiting improved mechanical properties. The comprehensive mechanical properties of the BHTSs were evaluated using finite element (FE) models constructed in ANSYS, which were validated by the experimental results. Light-weight numbers (LWNs) were used as an index to assess these properties. The simulation results were compared with the experimental data to validate the findings. The compression results indicated that the maximum load carried by each BHTS was very similar, with the highest bearing load being 32,571 N and the lowest being 30,183 N, resulting in only a 7.9% difference between them. In terms of the LWN-C values, the BHTS-1 exhibited the highest value at 318.51 N/g, while the BHTS-6 had the lowest value at 295.16 N/g. For the torsion and bending results, these findings suggested that increasing the bifurcation structure at the end side of the thin tube branch significantly improved the torsional resistance properties of the thin tube. For the impact characteristics of the proposed BHTSs, enhancing the bifurcation structure at the end of the thin tube branch significantly increased the energy absorption capacity and improved the energy absorption (EA) and the specific energy absorption (SEA) values of the thin tube. The BHTS-6 had the best structural design in terms of both the EA and SEA among all the BHTSs, but its CLE value was slightly lower than that of the BHTS-7, indicating slightly lower structural efficiency. This study provides a new idea and method for developing new lightweight and high-strength materials as well as designing more effective energy absorption structures. At the same time, this study has important scientific value in understanding how biological structures in nature exhibit their unique mechanical properties.

## 1. Introduction

Through billions of years of evolution, some biological structures already have excellent properties and ingenious frameworks to mitigate the collision and impact loads of the complex surrounding environments, protecting biological structures from being damaged [1,2,3]. Additionally, compared with conventional artificial protection structures, the biological structures possess excellent impact resistance properties and high energy dissipation efficiency [4,5,6,7,8]. Therefore, nature is an endless source of inspiration for scientists and engineers in different fields, where people can draw inspiration to design materials with better performance, by imitating the biological morphology, structure, and control principles [9,10,11]. In addition, biomimetics has already attracted worldwide attention in the field of lightweight design [12,13,14]. For example, inspired by biomimetic structures, such as spider webs, as a novel lightweight energy absorber, Zhang et al. created a fractal hierarchical hexagon structure and studied its energy absorption efficiency. The computational results revealed that both simple hierarchical and fractal structures present significant improvements in energy absorption over a single-wall nonhierarchical structure, offering a new route for designing novel lightweight energy absorbers with improved crash protection against impact [15]. Yang et al. extracted the structural characteristic of Odontodactylus scyllarus to design a novel lightweight bioinspired double-sine corrugated (DSC) sandwich structure to enhance the impact resistance, which significantly improved the structural crashworthiness and reduced the initial peak force greatly, compared with the regular triangular and sinusoidal corrugated core sandwich panels [16]. Ma et al. drew inspiration from the structural characteristics of bamboo, designing a biomimetic cylindrical structure to mimic the gradient distribution of vascular bundles and parenchyma cells. Furthermore, the buckling resistance of the biomimetic structure was compared with that of a traditional shell of equal mass under axial pressure by finite element simulations; it was found that the load-bearing capacity of the biomimetic shell increased by 124.8% [17]. Song et al. designed a variable thickness structure (VTS) inspired by the gradient of thickness and the internodal distance along the growth direction of bamboo and studied its energy absorbing performance. Meanwhile the structure was also optimized through a multiobjective optimization method and polynomial regression (PR) metamodel and compared with circular tube. The results showed that the optimal VTS was superior to the circular tube in terms of the energy absorption. Moreover, the crushing force efficiency increased by 7.48%, and the mass decreased by 19.3% [18].

Thin-walled designed structures are a highly promising material with a range of potential applications in various industries that require lightweight, high-strength, and energy-absorbing structures. In the automotive industry, this structure has been identified as an excellent energy-absorbing material for manufacturing safety components such as airbags and vehicle body frames. Similarly, in the aerospace sector, thin-walled designed structures could be used for manufacturing aircraft fuselages, landing gear, and other structural components for high-speed flying machines. Additionally, in the construction field, using this material can improve the thermal insulation properties and energy efficiency of buildings. Lightweight structures, especially thin-walled tube structures, are widely used in the aerospace, automotive, and transportation industries, for military equipment, and in other fields due to their light weight, low cost, and high energy-absorbing capabilities. The use of these structures has become an important trend in the development of modern industrial technology, as evidenced by recent research [19,20,21]. Further investigations have been conducted to explore their potential applications and capabilities [22,23]. Xiang et al. designed a new biomimetic bi-tubular thin-walled structure (BBTS) inspired from the internal structure of the lady beetle elytron and investigated its energy absorption performance. The results indicated that the thickness of the inner wall and the cross-sectional configurations significantly influenced the energy absorption of the structure. At the same time, the BBTSs showed an optimized crashworthiness behavior when the inner wall thickness was between 1.6 and 2.0 mm. Moreover, the optimized regular structure outperformed the original biomimetic topology in terms of the mechanical performance [24]. Ma et al. designed a bioinspired multicell corrugated tube for use in energy absorption and investigated the crashworthiness of thin-walled bioinspired multicellular corrugated tubes under an axial load numerically. The results showed that the multicell corrugated tubes had good crashworthiness [25]. Ha et al. proposed novel bioinspired fractal multicell circular (BFMC) tubes for energy absorption and investigated the crashworthiness performances of the proposed structures with different fractal orders and mass. The numerical results indicated that the specific energy absorption (SEA) increased with the fractal order, and the SEA of the second-order BFMC tube was 35.43% higher than that of the conventional multicell circular tube [26].

In recent years, different avenues have been explored by researchers to incorporate natural forms and functions into technological solutions through the increasingly popular approach of biomimicry in engineering design [27,28,29]. For example, bamboo-biomimetic tubes were developed by Fu et al. to enhance their energy absorption characteristics under axial crushing inspired by the microstructure of bamboo. The optimal rib configuration for improved crashworthiness performance was identified, using finite element analysis to investigate the effects of the rib shape and number [30]. Similarly, Xiao et al. investigated the crashworthiness of horsetail-biomimetic thin-walled structures under axial dynamic loading by evaluating six HBTSs with different cross-section configurations, using nonlinear finite element simulations. An ensemble metamodeling technique was employed to identify the Pareto optimum designs for all six HBTSs [31]. Xie et al. presented an evaluation method for the energy-absorption characteristics of thin-walled composite structures with random uncertain parameters by utilizing the finite element method to accurately simulate the initial peak load, load–displacement curve, and SEA value [32]. Biomimetic design methods were also used by Zou et al. (2016) to increase the axial and lateral energy absorption of thin-walled tubes by introducing the structure of bamboo. The biomimetic design was shown to enhance the specific energy absorption (SEA) of the tubes through numerical examples of biomimetic structures under axial/lateral impacts solved with nonlinear finite element method simulations [33]. Moreover, Patel et al. studied the crashworthiness performance of monolithic and coaxial multiwall frusta tube structures under quasistatic axial loading. They employed a nonlinear finite element analysis code LS-DYNA to simulate a series of layered configurations and utilized the optimization technique GRA (Grey Relational Analysis) to obtain better combinations of multiwall layered structures [34]. These studies provide valuable insights into the different methods and models that can be used to enhance the performance of technological solutions based on natural forms and functions through biomimicry in engineering design. The proposed approach in this paper builds on these previous works by introducing a novel biomimetic-bamboo thin-walled structure and utilizing topology optimization to further optimize its crashworthiness performance.

The hierarchical structure of lotus leaves inspired the design of the biomimetic hierarchical thin-walled structures (BHTSs) in this study. Through finite element analysis, the static comprehensive mechanical properties of these BHTSs were investigated. To verify the simulation findings, the proposed structures were fabricated using Stereolithography Apparatus (SLA) technology and R4600 resin material. The mechanical properties of the various BHTSs were then experimentally determined and compared with the simulation results. Moreover, the effect of the BHTSs’ on the crashworthiness performance was also examined. The results of this research hold great significance for the development of lightweight structures, particularly thin-walled structures in aerospace engineering, robot arms, and other tubular structures. By mimicking the hierarchical structure of lotus leaves, the BHTSs were designed to possess superior mechanical properties, such as a high strength-to-weight ratio, energy absorption capacity, and impact resistance. These properties are highly desirable for applications where weight reduction and structural stability are crucial factors. Furthermore, this study demonstrates the effectiveness of using finite element analysis and 3D printing technology in designing and fabricating BHTSs. The utilization of these techniques provides an efficient and cost-effective way to optimize the design and improve the mechanical properties of lightweight structures. Overall, the findings of this study have important implications for the development of lightweight materials and structures in different fields. The application of bioinspired designs and advanced manufacturing technologies could lead to the creation of more efficient and sustainable structures that can meet the increasing demands of modern engineering applications.

## 2. Materials and Methods

### Crashworthiness Indicators

Numerous crashworthiness indicators play an essential role in evaluating thin-walled structures’ crashworthiness. In this study, several indicators were defined to evaluate the performance of the tested materials under impact loading conditions. These indicators include the total energy absorption (*EA*), specific energy absorption (*SEA*), peak crushing force (*PCF*), and crash load efficiency (*CLE*). These indicators consider various factors that contribute to the overall performance of the structure during a crash event. The *EA* measures the amount of energy absorbed by the structure, while the *SEA* represents the energy absorbed per unit mass. The *PCF* indicates the maximum force generated during the deformation process, and the *CLE* quantifies the ability of the structure to absorb energy under real-world crash scenarios. The combination of these indicators provides a comprehensive evaluation of the thin-walled structure’s crashworthiness performance, which is critical for ensuring passenger safety in the automotive industry, among other applications [35]. The performance of the BHTS tubes was evaluated using the crashworthiness criteria developed by Chen et al. [36] and Xu et al. [37].

The energy absorption (*EA*) refers to the total amount of energy that is absorbed by a structure during its deformation under impact. This value is obtained by integrating the load–displacement curve during the loading process. Additionally, a higher energy absorption (*EA*) indicates the better crashworthiness of the structure. The *EA* is calculated by:

(1) $$EA=\int_{0}^{d}F\left(x\right)dx,$$

where *x* is the instantaneous crushing displacement, *F*(*x*) is the crushing force, and *d* is the effective crushing displacement.

*SEA* is the energy absorption per unit mass, which can be defined by:

(2) $$SEA=\frac{EA}{M},$$

where *M* is the total mass of the structure, and the *SEA* is used to estimate the energy absorption efficiency of structures with different materials and weights. Therefore, the higher the *SEA*, the better the capability of the energy absorption.

The peak crushing force *PCF* is the maximum of *F*(*x*), which is another important crashworthiness indicator for energy absorbers. Additionally, the *PCF* is one of the injury-based metrics, a large *PCF* will trigger large deceleration, potentially causing a serious injury of passengers. Therefore, from the safety design viewpoint, the *PCF* needs to be reduced as much as possible or constrained to a certain extent.

The mean crushing force (*MCF*) is defined as the average compression force resisted by the energy absorber during the total plastic deformation process. It is formulated by

(3) $$MCF=\frac{EA(d)}{d},$$

where *E*(*d*) represents the total energy absorbed at a certain impact distance, and *d* is the impact distance.

Lastly, the *CLE* is used to measure the load uniformity, indicating the constancy of the crushing force during impact. The *CLE* can be expressed mathematically as:

(4) $$CLF=\frac{MCF}{PCF}.$$

Obviously, the higher the *CLE* value, the more efficient the structure.

## 3. Results and Discussion

### 3.1. Biomimetic Tube Design

**Figure 1:**
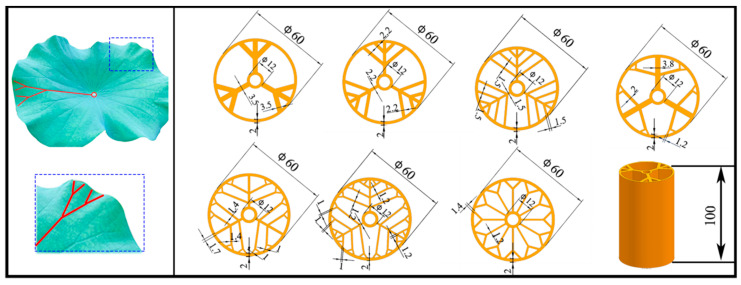
The designed models of biomimetic structures inspired by a lotus leaf (unit: mm).

This section describes the structural features of the lotus leaf and how they were utilized to design the biomimetic wall tubes. The lotus leaf has a radial network of main veins and branch veins that support the weight and external load of the leaf, ensuring its overall stiffness and plane ductility. The main veins bear the main load, while the branch veins distribute the stress near the edge more uniformly, further strengthening the bearing capacity of the edge near the lotus leaf. By introducing these structural features into the design of thin-walled tubes, it has been found that they can effectively improve their mechanical properties. The authors of this study propose a series of BHTSs based on the structural characteristics of the lotus leaf and evaluate their compression, torsion, bending, and impact characteristics through simulation analysis. Figure 1 shows the designed models of the biomimetic hierarchical thin-walled structures inspired by a lotus leaf. The BHTS-1, BHTS-2, and BHTS-3 designs all use the three main veins of the lotus leaf as the internal support, with several branching loads on each side of the main vein. The BHTS-1 has only one load, while the BHTS-2 and BHTS-3 increase the number of loads. This design can improve the load-bearing capacity of thin-wall tubes by increasing the number of branching loads, while maintaining a simple structure. The BHTS-4, BHTS-5, and BHTS-6 have additional branching layers on each branching load compared to the previous designs, thereby increasing the number of internal supporting layers and improving the stiffness and bending resistance of the thin-wall tubes. The BHTS-7 uses a different internal support method, where evenly distributed radial veins of the lotus leaf serve as internal supports, and a single load is branched on each dispersed vein. This design increases the number of internal support points, making the distribution of stress more uniform, thereby improving the overall strength and stability of the thin-walled tubes.

**Figure 2:**
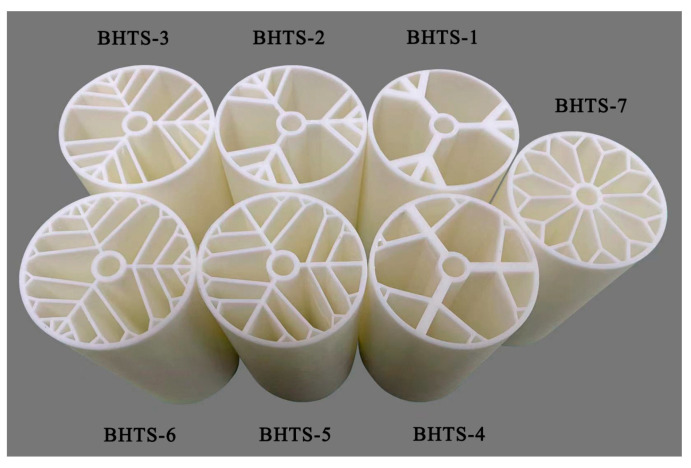
Additively manufactured tubes.

To assess the mechanical properties of the additively manufactured tubes, the commercial finite element software ANSYS Workbench 18.2 was utilized to simulate various conditions, including compression, torsion, and bending. R4600 resin material was used in the 3D printing process to manufacture the actual samples; so, its properties were incorporated into all simulations. The manufacturer provided the material properties in Table 1 for reference purposes. As shown in Figure 2, the additively manufactured tubes are highly complex structures, and their mechanical properties are critical for their performance in various applications such as the aerospace and medical industries. The simulations were designed to evaluate the tubes’ response under different loads and to provide insights into their structural behavior, which can aid in the design and optimization of these components.

The lightweight efficiency of each BHTS was evaluated using lightweight numbers (LWN) as a metric, which can be formulated based on the methods proposed by Li et al. [38] and Bukner et al. [39]:

(5) $$\mathrm{LWN}=\frac{\mathrm{Maxload}}{\mathrm{Weight}}.$$

where LWN-C is the compression, LWN-T is the torsion, and LWN-B is the bending.

In this study, the mechanical behavior of the proposed structure was evaluated under different loading conditions, including compression, torsion, and three-point bending using the numerical method of finite element analysis (FEA). FEA is considered an appropriate solution for simulating and analyzing complex systems, enabling the prediction of the response and deformation of the structure under various loading scenarios. Significant progress has been achieved in various fields through previous studies based on FEA, such as the optimization of aircraft and spacecraft structures for improved efficiency and safety, and the development of new materials for various applications by investigating material behavior under different conditions.

The material used in the simulation was consistent with the material used in the experiment, as shown in Table 2. It was assumed that the material was homogeneous, and its density, Young’s modulus, and Poisson’s ratio were uniform throughout the material. It was also assumed that the material exhibited linear elastic behavior under small strains. The tensile strength of the material was assumed to be measured in the longitudinal direction and was applicable even when the stress direction was different from the longitudinal direction.

### 3.2. Compression Properties of the Proposed BHTSs

**Figure 3:**
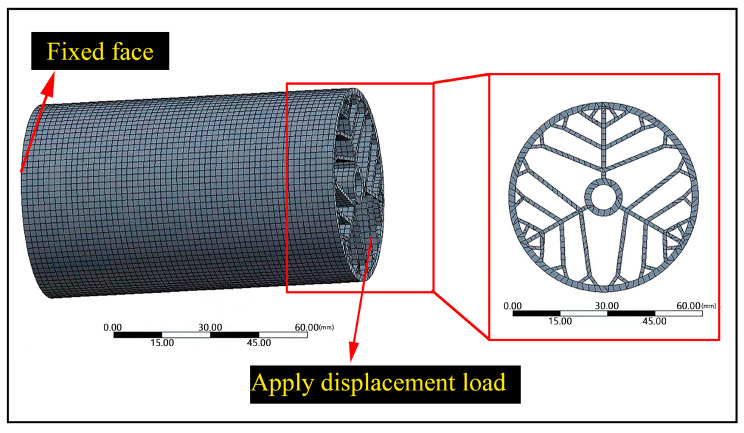
Compression finite element model of the BHTS-6.

The compressive properties of the BHTSs were investigated through both finite element simulations and experimental methods. A compression finite element model was designed for the BHTS-6 as a case study, which is illustrated in Figure 3.

To replicate the compression experiment, the bottom surface of the BHTS-6 was fixed, and a vertical load was applied along its central axis. Concurrently, grid convergence tests were performed to ensure accuracy. The simulation was implemented using hexahedral elements (Solid186) with a 1 mm size. The FEA model comprised 143,124 nodes and 23,688 elements. The results from the simulation were analyzed by calculating the force–displacement curves, the maximum compressive loads, and the LWN-C.

**Figure 4:**
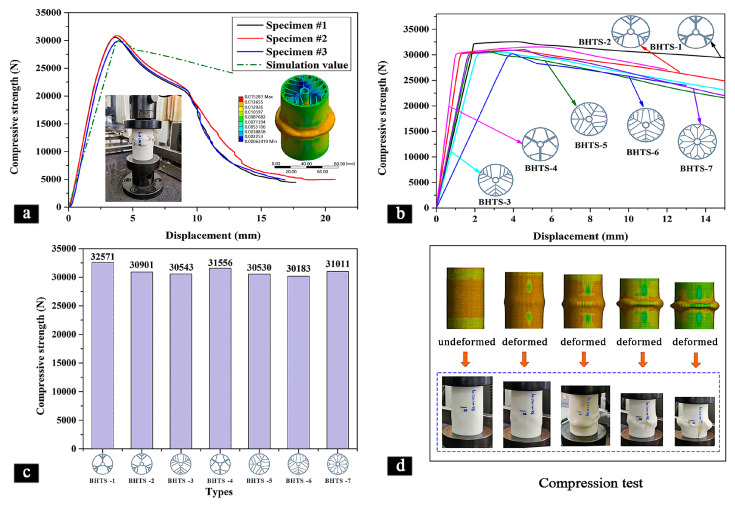
The compressive properties of the different BHTSs. (**a**) The comparison between the experimental and simulation results for the BHTS-6; (**b**) load–displacement simulation curves for the different BHTSs; (**c**) maximum compressive load of the BHTSs; (**d**) compression test.

Figure 4 displays the compression properties of the various BHTSs. As shown in Figure 4a, the compressive strength of the three samples of the BHTS-6 and one simulation model increased with the displacement and reached failure at approximately 3 mm of displacement, indicating that they all exhibited similar compression behavior. This suggests that the numerical simulation accurately captured the compression failure curve observed in the actual samples. Moreover, it can be observed from Table 2 that the actual compressive strength of the BHTS-6 was only 1.6% higher than the simulated compressive strength, which confirmed the accuracy and reliability of the FEM modeling approach and further supported its use in assessing various other performance characteristics of the BHTSs.

Figure 4b demonstrates that the compressive strength of the different BHTSs increased with an increase in the displacement. The compressive strength attained its maximum when the thin tube was destroyed, and then it gradually decreased. The BHTS-2 and BHTS-4 were disrupted around 1 mm displacement, while the BHTS-6 disruption had the largest displacement of 4 mm. The corresponding displacement of the rest of the BHTSs during destruction was close. The BHTS-1 exhibited significant compressive strength during destruction compared to the other BHTSs, indicating a better compression performance. The compression strength of the BHTS 1 was higher than the other BHTSs. This was mainly because the BHTS 1 has three thicker main veins and branching structures compared to the other BHTSs that had the same weight. This structure effectively improved its overall compression strength. In addition, the BHTS 1 experienced less deformation when subjected to stress compared to the other BHTSs, reducing the twisting effect and improving the overall compression strength. These results indicate that the design and internal structure of the BHTS are crucial for its mechanical performance, and excellent mechanical properties can be achieved through proper design.

It is noteworthy that Figure 4c clearly shows the corresponding compression forces for each BHTS at the point of destruction. The figure reveals that the maximum load carried by each BHTS was very similar, with the highest bearing load being 32,571 N and the lowest being 30,183 N, a difference of only 7.9%. Furthermore, increasing the number of branches on the thin main vein slightly reduced the compression performance of the thin tube, as evidenced by the slight decrease in the maximum bearing load observed from the BHTS-1 to the BHTS-3. When comparing the BHTS-1 to the BHTS-4, the BHTS-2 to the BHTS-5, and the BHTS-3 to the BHTS-6, the latter exhibited slightly lower bearing capacity but with only a small difference in the bearing load. This suggests that increasing the bifurcation structure on the thin tube branch also decreased the compressive strength of the thin tube, but its impact was limited.

Figure 4d illustrates both the simulated and experimental compression destruction process for the BHTSs. The figure shows that the results from the simulation model matched well those obtained experimentally. Table 2 compares the compressed data between the experiment and the simulation, revealing the close proximity of the LWN-C values for each BHTS. The highest LWN-C value was observed for the BHTS-1 at 318.51 N/g, while the lowest LWN-C value was measured for the BHTS-6 at 295.16 N/g, with only a 7.9% variation between them.

### 3.3. Torsion Properties of the Proposed BHTSs

The structures were fixed with six degrees of freedom at one end and subjected to angular displacement on the other end to induce torsion. The reaction torque of the end face was fixed to determine the torsional load based on the torsion angle. The number of grid nodes and elements used in the torsion simulation was identical to that of the compression simulation.

**Figure 5:**
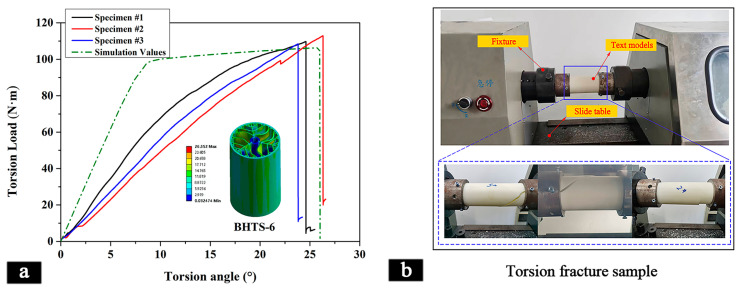
Torsion properties of the different BHTSs. (**a**) Torsional strength between the experimental and the simulation results for the BHTS-6; (**b**) torsion properties test.

Figure 5a displays the torque–torque angle curve for three samples of BHTS-6 and the corresponding theoretical model. The curves show that the torque increased with the increasing corner angle until it reached its maximum value, after which it rapidly declined. According to Table 3, the difference between the maximum torque observed in the actual samples and the theoretical model was only 3.7%. Additionally, the LWN-T values were also very close, with the simulated value being only 3.5% smaller than the experimental value. These findings suggest that the theoretical models provided an effective means of simulating the behavior of BHTSs, serving as a reliable alternative to physical testing.

The simulation curves demonstrate that all the BHTSs exhibited an increase in torsional load as the torsion angle increased, with sharp rises observed within the first 0–5° of torsion. Subsequently, slight damage occurred at 5–10°, and the rate of torsional load increase slowed down. Ultimately, all the BHTSs failed when the torsion angle reached approximately 25°. Notably, the BHTS-4 exhibited the highest maximum torsional load and the best torsional properties, while the BHTS-7 showed the lowest maximum torsional load and had inferior torsional resistance.

Table 3 illustrates the maximum torsional loading of the different BHTSs. The BHTS-3 and BHTS-7 had poor torsional properties with small maximum torsional loads of 80.15 N·m and 77.61 N·m, respectively, compared to the other BHTSs. In contrast, the BHTS-4 exhibited the highest maximum torsional load of 134.33 N·m. Analysis of the graph shows that the maximum torsional load of the BHTSs decreased with the increase in the number of branch layers inside the thin tube. This was because more branch layers led to a decrease in the cross-sectional area of the tube, making it more prone to failure under torsional forces. The uneven stress distribution within the structure also contributed to the reduction in the torsional properties. However, closer examination revealed a significant improvement in the maximum torsional loading of the BHTS-1, BHTS-2, and BHTS-3 compared to their internal structure, despite an increased number of branch layers. For example, BHTS-4 showed a 23.2% improvement in the maximum torsional loading over BHTS-1. Similarly, BHTS-5 exhibited a 36% increase in maximum torsional load when compared to BHTS-2, and BHTS-6 had a 32.7% increase in maximum torsional load when compared to BHTS-3. Increasing the bifurcation structure at the terminal side of thin tube branches greatly improved their torsional characteristics. Optimizing the bifurcation structure reinforced the thin tube and provided additional support, leading to the improved resistance to torsional forces. A well-designed bifurcation structure improves the stress distribution, increasing the cross-sectional area of the tube and enhancing its ability to resist torsional forces. Figure 5b demonstrates the torsional performance test of the sample model, resulting in its destruction due to excessive twisting. Improving the bifurcation structure is critical to achieving lightweight and high-strength materials with improved torsional properties, particularly for aerospace and biomedical engineering applications.

### 3.4. Bending Properties of the Proposed BHTSs

**Figure 6:**
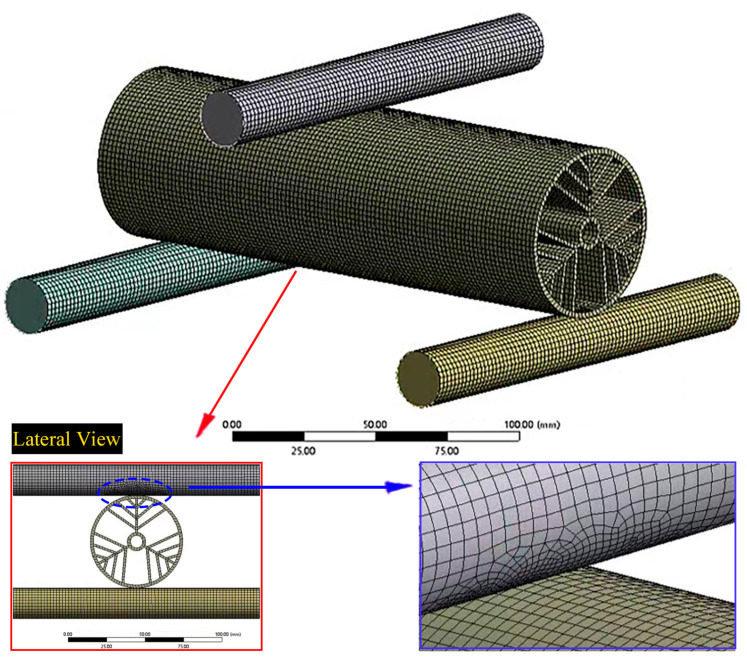
Bending finite element model of the BHTS-6.

For the bending simulations, the two ends of the structure were fixed and considered as rigid bodies, while the middle part was also a rigid body but subjected to a displacement load with 10 steps and a step length of 0.1 mm. The finite element model shown in Figure 6 used a shell 181 cell type and had a refined mesh size in the contact part. This type of simulation can help to understand the mechanical behavior of the BHTSs under bending loads and could be useful for further analysis or optimization of the design of such structures.

**Figure 7:**
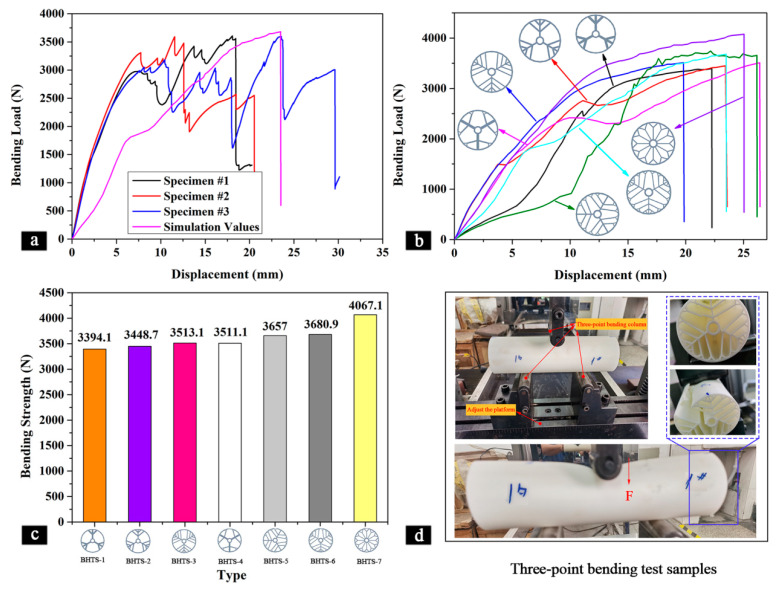
The three-point bending properties of the different BHTSs. (**a**) The comparison between the experimental results and the simulation for the BHTS-6; (**b**) bending-load–displacement simulation curves for different BHTSs; (**c**) maximum compressive load of the BHTSs; (**d**) bending test.

Figure 7 presents the three-point bending properties of the different BHTSs, and specifically, Figure 7a shows the three-point bending characteristic curves for three samples of the BHTS-6 and one simulation model. It can be observed that the four curves had a similar overall trend, with the bending load increasing as the displacement increased. Eventually, when the displacement reached a certain value, the bending load dropped sharply, indicating failure and damage to the BHTSs. Table 4 shows that the bending failure curve obtained from the numerical simulations agreed well with the one obtained from the actual samples. This suggests that the finite element model used in the simulation was accurate and could provide reliable results that were consistent with the experimental data. Such validation of the numerical model was crucial for ensuring its usefulness in the further analysis and optimization of BHTSs under bending loads.

Figure 7b shows the bending-load–displacement simulation curves for the different BHTSs. It is vividly seen that the bending-load–displacement curve showed the same trend for all the BHTSs. In general, the bending load of the BHTSs increased with the increase in the displacement, and when the displacement reached a certain value, the damage occurred, the bending load dropped sharply, and the thin tube failed. It can also be found that the BHTS-3 failed first at a displacement of 20 mm. Compared to the other BHTSs, the corresponding displacement was relatively large when the BHTS-4 and BHTS-5 failed, suggesting that they failed later. The same displacement corresponded to the disruption of the BHTS-2 and BHTS-6. However, the maximum bending load of the BHTS-6 was larger than the BHTS-2, and its bending characteristics were better than the BHTS-2. It is worth noting that the corresponding maximum bending load was the highest when the BHTS-7 failed.

Figure 7c clearly shows the flexural strength of the different BHTSs. It can be seen that the BHTS-7 had the greatest flexural strength of 4067.1 N. In addition to the BHTS-7, the remaining BHTSs had close flexural strength, and among them, the BHTS-1 had the lowest flexural strength of 3394.1 N. The BHTS-6 had the largest flexural strength of 3680.9 N, which was 8.4% higher than the BHTS-2. In addition, comparing the BHTS-1 and BHTS-2, the BHTS-2 was 1.6% higher than the BHTS-1. Comparing the BHTS-2 and BHTS-3, the latter was 1.9% higher than the former. It can be observed that the bending strength of the tested structures exhibited an increasing trend. This was because the BHTS-2 had one layer more of branch vein than the BHTS-1, and the BHTS-3 added another layer of branch vein compared to the BHTS-2, indicating that increasing the number of branch layers in the thin tubes improved the flexural strength of thin tubes to some extent. Comparing the BHTS-1 with the BHTS-4, the BHTS-4’s bending strength was 3.4% stronger than the BHTS-1. Comparing the BHTS-2 with the BHTS-5, the latter was 6% stronger than the former. Comparing the BHTS-3 and BHTS-6, the latter was 4.8% higher than the former. This was because relative to the BHTS-1, BHTS-2, and BHTS-3, the BHTS-4, BHTS-5, and BHTS-6 all increased the bifurcation structure at the end side of the branch vein. This indicates that increasing the bifurcation structure at the terminal side of the thin tube branch improved the flexural strength of the thin tube. In summary, the flexural properties of the BHTS-6 were optimal compared to the BHTSs of other designs in the three-point bending experiments, but there was still a gap with the BHTS-7 in the flexural properties. The bending strength of the BHTS-7 was 10.5% higher than that of the BHTS-6. The BHTS-7 had a higher bending load than the other BHTSs, mainly for two reasons. Firstly, the internal support and branching structure in the BHTS-7 was designed with more complexity, providing better bending stiffness and durability. These support and branching structures can evenly distribute the load and transfer it to the tube wall when subjected to bending loads, reducing the stress concentration on the tube wall and improving the strength and stability of the overall tube material. Secondly, the support and branching structures in the BHTS-7 were optimized and adjusted in different directions to maximize the mechanical performance of the tube material. For instance, in the bending direction, the support structure in the BHTS-7 was optimized to be longer and denser, increasing the bending strength of the tube material and offsetting some external forces, thereby achieving better dispersion and balance of the bending loads.

Figure 7d shows the actual bending performance test results. One can see that the final thin tube broke and failed.

### 3.5. Impact Characteristics of the Proposed BHTSs

**Figure 8:**
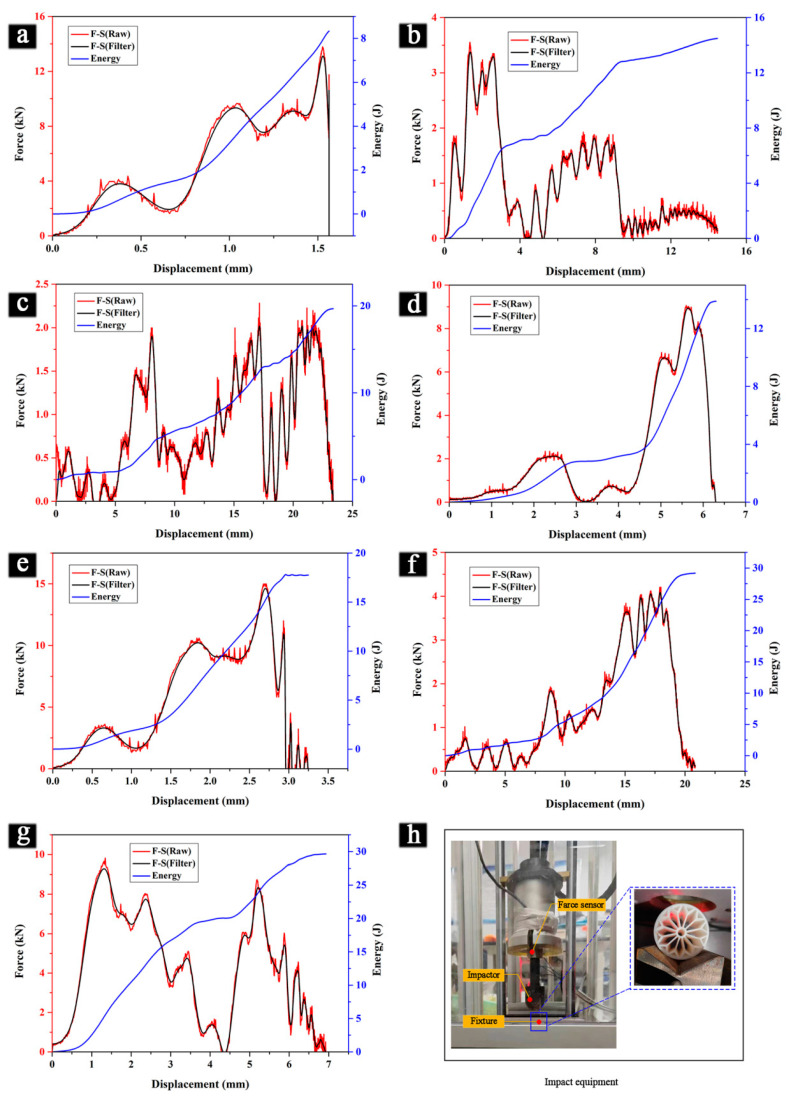
Force–displacement and energy–displacement curves of each BSs. (**a**) BHTS-1; (**b**) BHTS-2; (**c**) BHTS-3; (**d**) BHTS-4; (**e**) BHTS-5; (**f**) BHTS-6; (**g**) BHTS-7; (**h**) experimental equipment.

To assess the crashworthiness behaviors of the proposed structures, impact resistance tests were conducted using a drop-hammer impact testing machine (DIT302A-TS, Shenzhen Wance, Shenzhen, China). The biomimetic structures were subjected to impacts of a specified mass and shape from a set height. The impact resistance of the samples was evaluated based on the experimental results obtained. Table 5 provides details on the technical parameters of the drop-hammer impact testing machine used in this study. Figure 8a–g illustrate the force–displacement and energy–displacement curves for the different biomimetic lightweight tube samples, while Figure 8h shows the drop-hammer impact testing machine itself. Through conducting these tests, the effectiveness of the proposed structures in withstanding the impact forces and the absorbing energy was evaluated. These findings have important implications for developing durable and reliable structures that can withstand different types of loading conditions and impacts.

The primary veins of the lotus leaf are robust and arranged in a rib-like pattern. The secondary veins are deeply intertwined to form a grid of uniformly sized and structurally stressed cells. Each cell is connected by triangles, which provide strong stability and enable the entire structure to function as a cohesive unit. In addition to their role in nutrient transport, the leaf veins also support the weight and external load of the leaf, ensuring its overall stiffness and flexibility. As such, the distribution and size of the veins play a critical role in the bearing capacity and maintaining the leaf’s shape. To minimize the experimental errors, three samples were produced for each thin-tube structure, and impact tests were conducted on each sample separately. The results of the energy absorption performance evaluation parameters, including the EA, SEA, MCF, PCF, and CLE values, are summarized in Table 6.

Table 6 reveals that the BHTS-6 and BHTS-7 exhibited significantly higher EA and SEA values than the other BHTSs, and these two samples displayed a similar energy absorption capacity. This suggests that they possess robust energy absorption capabilities. On the other hand, the BHTS-1 displayed the lowest EA and SEA values, namely 8.37 J and 82.03 J, respectively, indicating poor energy absorption capacity. However, its high CLE value signified a good load uniformity. Additionally, the BHTS-2 and BHTS-4 had similar EA and SEA values, indicating little difference in energy absorption capacity between them. For a more detailed comparison of the energy absorption capacity of the different BHTSs, refer to Table 7. As depicted in Table 7, the EA and SEA values of the BHTS-1, BHTS-2, and BHTS-3 exhibited a gradual increase. This is attributed to the fact that the BHTS-2 has an additional layer of branch veins compared to the BHTS-1, while the BHTS-3 has an extra layer of branch veins compared to the BHTS-2. These results indicate that the energy absorption capacity of the thin tube structure increases with the increase in the number of layers in the branch vein structure.

Based on the comparison between the BHTS-1 and BHTS-4, it can be observed that the latter had significantly higher EA and SEA values by 64.4% than the former. This is attributed to the fact that each terminal branch of the BHTS-4 had one additional layer of bifurcation compared to the BHTS-1. Similarly, the BHTS-5 also outperformed the BHTS-2 in terms of the EA and SEA values by 23.8%. Additionally, the BHTS-6 exhibited a 53.1% and 53.3% increase in the EA and SEA values, respectively, compared to the BHTS-3. This is due to the same reason, i.e., the BHTS-6 had one extra layer of bifurcation in every terminal branch in contrast to the BHTS-3. These comparisons demonstrate that enhancing the bifurcation structure at the end of the thin tube branch increased the energy absorption capacity and improved the EA and SEA values of the thin tube. This improvement is attributed to the biomimetic design of the internal support structure and branching load distribution, which allows for better stress distribution and resistance to deformation. Specifically, the internal support structure of the BHTS-6 and BHTS-7 designs provided additional stiffness and stability to the thin-wall tubes, while the branching load distribution helped to distribute the stress more evenly and prevent localized failure. Based on the results of the impact test, it can be concluded that the structural design of the BHTS-6 was superior to the other BHTSs in terms of both the EA and SEA. It is interesting to note that the EA and SEA values of the BHTS-6 and BHTS-7 were similar. However, the BHTS-6 had a CLE value that was 20.9% lower than that of the BHTS-7, which indicates that its structural efficiency was slightly lower than that of the BHTS-7.

### 3.6. Summary

A novel biomimetic hierarchical thin-walled structure (BHTS) design inspired by the lotus leaf was proposed in this research, which offers several advantages over existing methods for designing energy absorption structures. The BHTS design has unique features that enable highly controllable energy absorption compared to other methods. Lightweight composite materials and hierarchical structure were used in our design, which allowed for a lightweight and compact design suitable for applications where weight and space constraints are important considerations. Additionally, the BHTS design can be customized to meet specific requirements, providing greater flexibility in design. By highlighting these benefits, it is believed that the proposed method offers improved performance and practical implications compared to other methods in the field.

From a theoretical perspective, the use of lightweight numbers (LWNs) as an index to assess the mechanical properties of biomimetic hierarchical thin-walled structures (BHTSs) was introduced in this study. This approach enabled a more accurate evaluation of the performance of the lightweight materials and structures for applications such as the crashworthiness. The effectiveness of using ANSYS to simulate and analyze the behavior of additively manufactured tubes under various loading conditions, including compression, torsion, bending, and impact, was also demonstrated. Valuable insights into the structural behavior and deformation mechanisms of these tubes were provided through simulations. From an experimental standpoint, the physical samples of BHTSs were fabricated using 3D printing with R4600 resin material, and tests were conducted to validate the accuracy of the simulation results, including the compression, torsion, bending, and other forms of loading. The findings indicate that incorporating bioinspired features in the design of thin-walled structures can significantly improve their energy absorption capacity and mechanical properties. This study presents a new approach to designing high-performance lightweight materials and structures with effective energy absorption capabilities, which can contribute to the development of innovative solutions in various industrial sectors.

## 4. Conclusions

This study systematically investigated the mechanical properties and crashworthiness of a series of biomimetic hierarchical thin-walled structures inspired by a lotus leaf. The experimental results were validated by finite element modeling (FEM) simulations and theoretical analysis. Based on our findings:

1. In the compression test, each BHTS had a similar maximum load-bearing capacity, with a 7.9% difference between them, with the highest bearing load being 32,571 N and the lowest being 30,183 N. The BHTS-1 showed a better performance in compression and significant compressive strength during destruction compared to the other BHTSs. Increasing the bifurcation structure on the thin tube branch decreased the compressive strength of the thin tube.
2. In the torsion test, the theoretical models accurately simulated the behavior of the BHTSs with only a 3.7% difference between the maximum torque observed in the actual samples and the theoretical model. The BHTS-4 had the best torsional properties, while increasing the branch layers decreased the maximum torsional load. The BHTS-7 showed inferior torsional resistance. Enhancing the bifurcation structure at the terminal side of the thin tube branch significantly improved torsional properties.
3. In the bending test, increasing the branch layers improved the flexural strength of the thin tubes to some extent. The BHTS-7 exhibited the highest flexural strength, while the BHTS-1 had the lowest. Enhancing the bifurcation structure at the terminal side of the thin tube branch improved the flexural strength. The BHTS-7 had a 10.5% higher bending strength than the BHTS-6 due to its more complex support and branching structures.
4. In the impact test, enhancing the bifurcation structure at the end of the thin tube branch increased the energy absorption capacity and improved the *EA* and *SEA* values. The BHTS-6 and BHTS-7 showed robust energy absorption capabilities, with significantly higher *EA* and *SEA* values than the other BHTSs. Compared to the BHTS-3, the BHTS-6 showed a 53.1% increase in the *EA* value and a 53.3% increase in the *SEA* value.

## Figures and Tables

**Table 1:** Material properties of the R4600 resin material.

Material Name	R4600 Resin Material
Density (g/cm^3^)	1.3
Young’s modulus (MPa)	2600
Poisson’s ratio	0.42
Elongation at break	10%
Tensile strength (MPa)	47

**Table 2:** Comparison between the compression data from the experiments and the simulations.

Type	Simulation/Test Values	Mass (g)	Maximum Compressive Load (N)	LWN-C (N/g)
BHTS-1	Simulation values	102.26	32,571	318.51
BHTS-2	Simulation values	102.26	30,901	302.18
BHTS-3	Simulation values	102.26	30,543	298.68
BHTS-4	Simulation values	102.26	31,556	308.59
BHTS-5	Simulation values	102.26	30,530	298.55
BHTS-6	Specimen #1	102.53	30,568.24	298.14
	Specimen #2	102.47	30,852.55	301.09
	Specimen #3	102.31	29,883.65	292.09
	Simulation values	102.26	30,183	295.16
BHTS-7	Simulation values	102.26	31,011	303.26

**Table 3:** Comparison between the torsion data from the experiments and simulations.

Type	Simulation/Test Values	Mass (g)	Maximum Torsional Load (N·m)	LWN-T (N·m/g)
BHTS-1	Simulation values	102.26	109.05	1.07
BHTS-2	Simulation values	102.26	90.07	0.88
BHTS-3	Simulation values	102.26	80.15	0.78
BHTS-4	Simulation values	102.26	134.33	1.31
BHTS-5	Simulation values	102.26	122.45	1.20
BHTS-6	Simulation values	102.53	106.36	1.04
	Specimen #1	102.43	112.91	1.10
	Specimen #2	102.41	108.44	1.06
	Specimen #3	102.39	109.73	1.07
BHTS-7	Simulation values	102.26	77.61	0.76

**Table 4:** Comparison between the bending data from the experiments and the simulations.

Type	Simulation/Test Values	Mass (g)	Maximum Bending Load (N)	LWN-B (N/g)
BHTS-1	Simulation values	170.56	3394.1	19.90
BHTS-2	Simulation values	170.56	3448.7	20.22
BHTS-3	Simulation values	170.56	3513.1	20.60
BHTS-4	Simulation values	170.56	3511.1	20.59
BHTS-5	Simulation values	170.56	3657	21.44
BHTS-6	Simulation values	170.56	3680.9	21.58
	Specimen #1	171.32	3607.04	21.05
	Specimen #2	170.89	3590.37	21.01
	Specimen #3	171.27	3597.81	21.01
BHTS-7	Simulation values	170.56	4067.1	23.85

**Table 5:** Technical parameters of the DIT302A-TS.

Model	DIT302A-TS
Maximum impact energy (J)	300
Maximum sample diameter (mm)	100
Hammer lifting speed (m/min)	8.0
Height measurement error (mm)	≤±10
Lift hammer height range (mm)	300–2000

**Table 6:** Results of the impact test.

Types	Specimens	Mass (g)	Impact Distance (mm)	PCF (kN)	*EA* (J)	*SEA* (J/kg)	*MCF* (kN)	*CLE*
BHTS-1	#1	102.44	1.56	13.11	8.33	81.32	5.34	0.41
	#2	101.26	1.23	12.68	8.15	80.49	6.63	0.52
	#3	102.29	1.79	13.57	8.62	84.27	4.82	0.36
BHTS -2	#1	101.98	14.46	3.38	14.49	142.09	1.00	0.30
	#2	102.01	13.88	3.06	13.92	136.46	1.00	0.33
	#3	102.36	14.21	3.25	14.27	139.41	1.00	0.31
BHTS-3	#1	102.41	23.37	2.02	19.68	192.17	0.84	0.42
	#2	102.27	21.39	1.83	19.02	185.98	0.89	0.49
	#3	102.69	24.15	2.31	20.13	196.03	0.83	0.36
BHTS-4	#1	101.86	6.3	8.94	13.89	136.36	2.20	0.25
	#2	102.11	6.02	8.63	13.67	133.88	2.27	0.26
	#3	101.93	6.24	8.77	13.71	134.50	2.20	0.25
BHTS-5	#1	102.41	3.24	14.63	17.75	173.32	5.48	0.37
	#2	102.06	2.86	14.16	17.29	169.41	6.05	0.43
	#3	102.24	3.35	14.63	17.81	174.20	5.32	0.36
BHTS-6	#1	102.38	20.83	4.09	29.19	285.11	1.40	0.34
	#2	102.26	20.58	3.73	28.62	279.87	1.39	0.37
	#3	102.43	22.39	4.28	32.29	315.24	1.44	0.34
BHTS-7	#1	102.13	6.92	9.29	29.63	290.12	4.28	0.46
	#2	102.22	7.86	10.23	31.83	311.39	4.05	0.40
	#3	102.51	7.59	9.89	20.68	201.74	2.72	0.28

**Table 7:** Crashworthiness indicators of each BHTS (Mean ± SD).

Types	PCF (kN)	EA (J)	SEA (J/kg)	MCF (kN)	CLE
BHTS-1	13.12 ± 0.445	8.367 ± 0.237	82.03 ± 1.987	5.60 ± 0.932	0.43 ± 0.082
BHTS-2	3.23 ± 0.161	14.23 ± 0.287	139.32 ± 2.817	1.00 ± 0.000	0.31 ± 0.015
BHTS-3	2.05 ± 0.242	19.61 ± 0.558	191.39 ± 5.070	0.853 ± 0.032	0.42 ± 0.065
BHTS-4	8.78 ± 0.155	13.76 ± 0.117	134.91 ± 1.291	2.22 ± 0.040	0.25 ± 0.06
BHTS-5	14.47 ± 0.271	17.62 ± 0.284	172.31 ± 2.550	5.62 ± 0.384	0.39 ± 0.038
BHTS-6	4.03 ± 0.279	30.03 ± 1.975	293.41 ± 19.089	1.41 ± 0.026	0.35 ± 0.020
BHTS-7	5.24 ± 0.148	27.38 ± 5.906	276.75 ± 58.147	3.68 ± 0.842	0.38 ± 0.09

## Data Availability

Not applicable.
